# Supplementary material for: Bile-Based Cell-Free DNA Analysis Is a Reliable Diagnostic Tool in Pancreatobiliary Cancer
Source: Cancers (Basel). 2020 Dec 25;13(1):39. doi: 10.3390/cancers13010039 (PMC7818177; doi:10.3390/cancers13010039)
Supplement: Supplementary file 1 [file cancers-13-00039-s001.pdf]

cancers

Supplementary Materials

Bile-Based Cell-Free DNA Analysis Is a Reliable Diagnostic Tool in Pancreatobiliary Cancer

Caroline Driescher, Katharina Fuchs, Lena Haeberle, Wolfgang Goering, Lisa Frohn, Friederike V. Opitz, Dieter Haeussinger, Wolfram Trudo Knoefel, Verena Keitel and Irene Esposito

Table S1. Detailed information on patient collective

ID	Diagnosis	Tumor/ cyst localization	Bile available	Plasma available	Tissue available	CA 19-9 [U/ml]	Concordance in NGS results between tissue and plasma	Concordance in NGS results between tissue and bile	Concordance in NGS results between plasma and bile
1	Localized PDAC	Pancreatic head	yes	yes	yes	758.4	yes*	yes	yes*
2	IPMN	Pancreatic head	no	yes	no	25.9	-	-	-
3	Pseudocysts	Pancreatic head/ body/ tail	no	yes	no	-	-	-	-
4	Metastatic PDAC	Pancreatic head	yes	yes	yes	>10000	yes*	yes	yes*
6	Pseudocysts	Pancreatic head	no	yes	no	-	-	-	-
8	PSC	-	yes	yes	no	136.2	-	-	yes
9	Localized PDAC	Pancreatic head	yes	no	yes	34.4	-	yes	-
10	Metastatic CCA	Extrahepatic	yes	yes	yes	>10000	no	yes	no
11	Metastatic PDAC	Pancreatic head	no	yes	no	>10000	-	-	-
12	Metastatic PDAC	Pancreatic head	yes	yes	yes	>10000	yes	yes	yes
13	CBD obstruction	-	yes	no	no	167.0	-	-	-
14	Metastatic PDAC	Pancreatic head	no	yes	yes	809.7	yes*	-	-
15	Metastatic PDAC	Pancreatic head	no	yes	no	2298.0	-	-	-
16	Choledocholithiasis	-	yes	yes	no	-	-	-	yes
17	Metastatic PDAC	Pancreatic body	no	yes	yes	230.4	yes	-	-
18	Metastatic PDAC	Pancreatic head	no	yes	no	14.7	-	-	-
20	Localized PDAC	Pancreatic head	yes	yes	yes	123.6	no	yes	no
22	CBD obstruction	-	yes	no	no	-	-	-	-
23	Metastatic PDAC	Pancreatic head	yes	no	yes	188.4	-	yes*	-
25	Localized PDAC	Pancreatic head	yes	no	yes	8849.0	-	yes	-
27	Localized CCA	Intrahepatic	no	yes	yes	62.3	no	-	-
28	Metastatic PDAC	Pancreatic head	no	yes	yes	1476.0	yes	-	-
29	Metastatic PDAC	Pancreatic head	yes	yes	no	-	-	-	yes
31	Metastatic PDAC	Pancreatic head	no	yes	no	893.0	-	-	-
32	PSC	-	yes	no	no	16.9	-	-	-
33	Localized CCA	Extrahepatic	no	yes	yes	193.8	no	-	-
34	CBD obstruction	-	yes	no	no	-	-	-	-
35	CBD obstruction	-	yes	no	no	32.1	-	-	-
36	Metastatic PDAC	Pancreatic head	no	yes	yes	238.5	no	-	-
37	Metastatic PDAC	Pancreatic head	no	yes	no	22.6	-	-	-
38	Localized CCA	Intrahepatic	no	yes	yes	8.2	yes	-	-
40	Metastatic PDAC	Pancreatic head	yes	yes	yes	3508.0	yes*	yes	yes*
41	Localized PDAC	Pancreatic head	yes	yes	yes	1310.0	no	yes	no

42	Metastatic PDAC	Pancreatic head	no	yes	no	-	-	-	-
43	Localized PDAC	Pancreatic head	no	yes	yes	1574.0	no	-	-
44	Localized PDAC	Pancreatic head	yes	yes	no	117.1	-	-	yes*
46	Metastatic PDAC	Pancreatic tail	no	yes	yes	20.1	yes	-	-
47	Localized PDAC	Pancreatic head	no	yes	yes	47.6	no	-	-
48	Metastatic PDAC	Pancreatic body	yes	no	no	12.8	-	-	-
49	Metastatic PDAC	Pancreatic head	yes	no	no	>10000	-	-	-
50	CBD obstruction	-	yes	no	no	-	-	-	-
51	Metastatic PDAC	Pancreatic head	no	yes	yes	77.9	no	-	-
54	Chronic pancreatitis	-	yes	no	no	-	-	-	-
55	CBD obstruction	-	yes	no	no	264.5	-	-	-
56	Chronic pancreatitis	-	no	yes	no	-	-	-	-
57	Localized CCA	Extrahepatic	yes	no	no	87.1	-	-	-
58	Choledocholithiasis	-	yes	no	no	-	-	-	-
59	CBD obstruction	-	yes	no	no	-	-	-	-
60	Chronic pancreatitis	-	no	yes	no	-	-	-	-
61	Chronic pancreatitis	-	no	yes	no	-	-	-	-
62	Chronic pancreatitis	-	no	yes	no	-	-	-	-
65	Metastatic PDAC	Pancreatic head	yes	yes	yes	>10000	yes	yes	yes
66	PSC	-	yes	no	no	-	-	-	-
67	Pseudocysts	Pancreatic head	yes	no	no	-	-	-	-
68	Pseudocyst	Pancreatic head	no	yes	no	-	-	-	-
70	Choledocholithiasis	-	yes	no	no	-	-	-	-
71	Metastatic PDAC	Pancreatic tail	no	yes	no	8346.0	-	-	-
72	Localized PDAC	Pancreatic head	no	yes	yes	30.5	no	-	-
73	IPMNs	Pancreatic head/ body/ tail	no	yes	no	-	-	-	-
75	Chronic pancreatitis	-	no	yes	no	53.1	-	-	-
76	IPMN	Pancreatic body/ tail	no	yes	no	-	-	-	-
77	IPMNs	Pancreatic body/ tail	no	yes	no	9.4	-	-	-
79	Localized CCA	Extrahepatic	yes	yes	yes	303.1	no	yes	no
80	Metastatic CCA	Intrahepatic	no	yes	yes	7.4	yes	-	-
83	Metastatic PDAC	Pancreatic head	yes	no	no	>10000	-	-	-
84	Pseudocyst	Pancreatic head	no	yes	no	6.7	-	-	-
86	Metastatic PDAC	Pancreatic head	yes	yes	yes	293.0	yes	yes	yes
87	CBD obstruction	-	yes	no	no	-	-	-	-
88	Metastatic CCA	Intrahepatic	yes	no	yes	-	-	yes	-
89	Chronic pancreatitis	-	yes	no	no	-	-	-	-
90	CBD obstruction	-	yes	no	no	43.2	-	-	-

91	Metastatic CCA	Extrahepatic	no	yes	yes	2699.0	yes	-	-
92	PSC	-	no	yes	no	6.8	-	-	-
93	Choledocholithiasis	-	yes	no	no	-	-	-	-
94	Localized CCA	Intrahepatic	no	yes	yes	17.4	no	-	-
96	CBD obstruction	-	yes	no	no	-	-	-	-
98	Metastatic PDAC	Pancreatic head	yes	yes	no	1993.0	-	-	yes*
99	Metastatic PDAC	Pancreatic head/ body	no	yes	no	-	-	-	-
100	PSC	-	yes	no	no	360.2	-	-	-
101	Localized PDAC	Pancreatic head	no	yes	no	181.1	-	-	-

* NGS analysis revealed at least one concordant mutation

Table S2. Mutations detected in bile, plasma and/or tissue

	KRAS	*TP53*	*SMAD4*	*CDKN2A*	*PIK3CA*	*BRAF*	*GNAS*	*CDH1*	*IDH1*
**Localized CCA**									
27	-	-	-	-	-	-	-	p.Asp370Asn	-
33	p.Gly12Val	p.Arg248Gln	-	-	-	-	-	-	-
38	-	-	-	-	-	-	-	-	-
57	-	p.Val157Phe	-	-	-	-	-	-	-
79	-	p.Tyr163Cys	-	-	-	p.Val600Glu	-	-	-
94	-	-	-	-	-	-	-	-	p.Arg132His
**Localized PDAC**									
1	p.Gly12Asp	-	p.Arg445Ter	-	-	-	-	-	-
9	p.Gly12Asp	p.Tyr220fs	-	p. Arg108Tyr	-	-	-	-	-
20	p.Gly12Val	p.Tyr220Cys	p.Gln311Ter	-	-	-	-	-	-
25	p.Gly12Asp	-	-	-	-	-	-	-	-
41	p.Gln61Arg	-	-	-	-	-	-	-	-
43	p.Gly12Val	-	-	-	-	-	-	-	-
44	p.Gly12Asp<br>p.Gln61His	p.Arg273His<br>p.Arg280Ile	-	-	-	-	-	-	-
47	p.Gly12Arg	-	-	-	-	-	-	-	-
72	p.Gly12Arg	p.Arg249Met	-	-	p.Glu707Lys	-	-	-	-
101	p.Gly12Val	-	-	-	-	-	-	-	-
**Metastatic CCA**									
10	p.Gly12Val	-	-	-	-	-	-	-	-
80	-	p.Glu285Lys	p.Arg361His	-	-	-	-	-	-
88	p.Gly12Cys	p.Arg213Ter	-	p.Arg80Ter	-	-	-	-	-
91	-	-	-	-	-	-	-	-	-
**Metastatic PDAC**									
4	p.Gly12Cys	c.994-1G>A*	-	-	-	-	-	-	-
11	p.Gly12Asp	-	-	-	-	-	-	-	-
12	p.Gly12Asp	p.Lys164Glu	-	-	-	-	-	-	-
14	p.Gly12Asp	p.Ser166fs	p.Tyr260fs	-	-	-	-	-	-
15	p.Gly12Arg	-	-	-	-	-	-	-	-
17	-	-	-	-	-	-	-	-	-
18	p.Gln61Arg	-	-	-	-	-	-	-	-
23	p.Gly12Val<br>p.Gln61His	-	-	p.Arg80Ter	-	-	p.Arg201His	-	-
28	p.Gly12Asp	-	-	p.Val126Asp	-	-	-	-	-
29	p.Gly12Val	p.Gly245Ser	-	-	-	-	-	-	-
31	-	-	-	-	-	-	-	-	-

36	p.Gly12Asp	-	-	-	-	-	p.Arg201Cys	-	-
37	-	-	-	-	-	-	-	-	-
40	p.Gly12Val	p.Tyr107fs	-	-	-	-	-	-	-
42	-	-	-	-	-	-	-	-	-
46	p.Gly12Val	-	-	-	-	-	-	-	-
48	p.Gly12Asp	p.Arg175His	-	-	-	-	-	-	-
49	p.Gly12Arg	-	-	-	-	-	-	-	-
51	p.Gly12Val	p.Arg175His	-	p.His83Tyr	-	-	-	-	-
65	p.Gly12Val	-	-	-	-	-	-	-	-
71	p.Gly12Val	-	-	-	-	-	p.Arg201His	-	-
83	p.Gly12Val	-	-	p.Asp84Gly	-	-	-	-	-
86	-	-	-	-	-	-	-	-	-
98	p.Gly12Asp	p.Tyr234Asn	-	p.His83Tyr	-	-	-	-	-
99	-	-	-	-	-	-	-	-	-

*Mutation in splice site

Table S3. FFPE Tissue used for DNA Extraction.

Patient ID	Tissue type	Procedure
**Localized CCA**		
27	primary lesion liver	Left hemihepatectomy
33	primary lesion liver	Liver segment resection
38	primary lesion liver	Percutaneous liver biopsy
79	primary lesion liver	Liver segment resection
94	primary lesion liver	Right hemihepatectomy
**Localized PDAC**		
1	primary lesion pancreas	Pylorus-preserving pancreatoduodenectomy
9	primary lesion pancreas	Pylorus-preserving pancreatoduodenectomy
20	primary lesion pancreas	Pylorus-preserving pancreatoduodenectomy
25	primary lesion pancreas	Pylorus-preserving pancreatoduodenectomy
41	primary lesion pancreas	Total pancreatectomy
43	primary lesion pancreas	Pancreaticoduodenectomy
47	primary lesion pancreas	Pylorus-preserving pancreatoduodenectomy
72	primary lesion pancreas	Pylorus-preserving pancreatoduodenectomy
**Metastatic CCA**		
10	metastatic lesion lung	Lung segment resection
80	metastatic lesion liver	Percutaneous liver biopsy
88	metastatic lesion liver	Percutaneous liver biopsy
91	metastatic lesion falciform ligament	Exploratory laparoscopy
**Metastatic PDAC**		
4	metastatic lesion liver	Percutaneous liver biopsy
12	metastatic lesion liver	Percutaneous liver biopsy
14	metastatic lesion liver	Percutaneous liver biopsy
17	metastatic lesion liver	Exploratory laparoscopy
23	metastatic lesion liver	Percutaneous liver biopsy
28	metastatic lesion liver	Percutaneous liver biopsy
36	metastatic lesion peritoneum	Percutaneous laparoscopic peritoneal biopsy
40	primary lesion pancreas	EUS-guided forceps biopsy of pancreatic head
46	metastatic lesion liver	Percutaneous liver biopsy
51	primary lesion pancreas	Pylorus-preserving pancreatoduodenectomy
65	metastatic lesion liver	Percutaneous liver biopsy
86	metastatic lesion liver	Percutaneous liver biopsy
